# Clinical Experience with Octagam® 10 %, a solvent detergent virus inactivated intravenous immunoglobulin: a Canadian retrospective review of utilization

**DOI:** 10.1186/s13223-016-0138-9

**Published:** 2016-07-27

**Authors:** Stephen D. Betschel, Richard J. Warrington, Robert Schellenberg

**Affiliations:** 1 St Michael’s Hospital and the University of Toronto, 30 Bond St, Toronto, ON M5B 1W8 Canada; 2 Head, Section of Allergy & Immunology, Department of Internal Medicine, University of Manitoba, Winnipeg Health Science Center, GC319 820 Sherbrook St, Winnipeg, MB R3A 1R9 Canada; 3 St Paul’s Hospital and the University of British Columbia, 1081 Burrard St, Vancouver, BC V6C 1Y6 Canada

**Keywords:** Octagam^®^, IVIg, Indication

## Abstract

In Canada, intravenous immune globulin (IVIg) products are licensed for six disease indications, however it has been demonstrated that patients with a number of other conditions also benefit from IVIg. Here we report the routine clinical use of Octagam^®^ 10 % across three Canadian institutions. A total of 135 patients were treated with Octagam^®^, for conditions represented by five distinct indication groups. The results of this review indicate that Octagam^®^ has been well adopted and is prescribed to Canadian patients similar to other IVIg products. In alignment with current practices, 85 % of Octagam’s utilization was classified as appropriate based on Canadian IVIg guidelines.

## Background

### History of IVIg

The first reported success of antibody therapy was in 1890, when von Behring and Kitasato described how serum extracted from an infected rabbit could protect a naïve rabbit from the effects of diphtheria toxin [1]. With the advent of large-scale fractionation, and access to blood banks through the American Red Cross, the first immunoglobulin products became available in the 1940s [2]. These products became routinely used by both subcutaneous and intramuscular route to prevent and treat infections [2]. Intramuscular administration of immune serum globulin was later described as a successful therapeutic approach to treat a patient who had a primary immune deficiency (PID) in 1952 [3], and quickly became standard practice for the treatment of patients with antibody deficiency syndromes [2]. With improvements in manufacturing and purification processes, several European companies began producing serum immunoglobulin suitable for intravenous administration in the 1960s [2]. With fewer reported side effects and improved efficacy [4, 5], intravenous administration became the mainstay approach for serum immunoglobulin treatment. Today, commercially available intravenous human normal immunoglobulin (IVIg) products are prepared from pooled plasma from large groups of healthy blood donors [1], providing a diversity of antibodies. Since its use as a replacement therapy for PID patients, the clinical use of IVIg has expanded and has become an important treatment option in a wide spectrum of diseases. IVIg is used routinely in autoimmune and acute inflammatory conditions due to IVIg’s immune-modulatory and anti-inflammatory properties.

### Current Canadian landscape

In Canada, IVIg products are distributed by the Canadian Blood Services with the exception of Québec, where Héma Québec assumes this role. The products are distributed to Canadian blood banks within hospitals, at which point they are dispensed to patients as physicians prescribe them. IVIg is currently used as replacement therapy in primary and secondary immunodeficiencies. IVIg is also used as an immunomodulatory therapy in a number of autoimmune diseases. Among the 8 IVIg products available in Canada, there are six different approved indications (replacement and immunomodulatory), including primary and secondary immunodeficiencies (PID/SID)—each with several different primary pathologies, chronic B-cell lymphocytic leukemia (CLL), immune thrombocytopenic purpura (ITP), chronic demyelinating polyneuropathy (CIDP) and multifocal motor neuropathy (MMN) although it is used for a variety of other indications with a range of data to support its use. Although no single product in Canada is indicated for all of these conditions, Canadian hospitals stock a variety of IVIg products that tend to be used interchangeably. Canada is the highest per-capita consumer of IVIg products worldwide, and the usage of IVIg in Canada has been steadily increasing over the last two decades [6]. To help ensure IVIg use is in keeping with an evidence-based approach to the practice of medicine, the National Advisory Committee on Blood and Blood Products (NAC) and Canadian Blood Services developed evidence-based practice guidelines on the use of IVIg for various conditions [7–12]. The objectives of these guidelines were to examine the evidence for the use of IVIg in specific conditions and provide recommendations to physicians on the optimal utilization of IVIg therapy in various therapeutic areas.

### Differences between IVIg products

Various product characteristics contribute to the tolerability of the IVIg preparation including form (liquid versus lyophilized), type of stabilizer, product purity, osmolarity, concentration, IgG and IgA content, pH, osmolality, and sodium content [13, 14]. IVIg therapy is generally well tolerated [15–17], with most adverse events being mild, reversible infusion related events such as rigors, low-grade fever, headache, myalgia, backache, flushing, and nausea [17, 18]. The incidence of mild adverse events is reported to be between 5 and 15 % per infusion [19]. Although some IVIg products may have a lower incidence of drug-related adverse reactions than others, differences in reporting, and lack of comparative trial data makes this assertion difficult. Serious IVIg treatment-related adverse events are rarely reported, and are more common in patients with risk factors for poor prognosis [18, 19]. The safe and effective use of IVIg requires attention not only to product characteristics but also to administration issues and patient related risk-factors. It is critical that patients receiving IVIg therapy are carefully evaluated and monitored so that treatment can be optimized.

In terms of clinical efficacy, there are limited numbers of trials that directly compare two products head-to-head. The small number of studies that compared two IVIg products were primarily licensing studies and not studies designed to compare product efficacy [20–23]. Dhainaut et al. compared in vitro and in vivo biological and biochemical properties of five liquid IVIg preparations. The authors concluded that the origin of plasma and the type of IVIg purification processes both contribute to obtain a pharmaceutical product with distinct biochemical and physiological properties, although all IVIg preparations were found to be efficacious in various patients groups [24]. A Canadian consensus statement also considered different IVIg products to have comparable clinical efficacy [11]. However, some patients may tolerate one product better than another [25, 26].

### Efficacy, tolerability and safety of Octagam^®^

Octagam^®^, a liquid ready-to-use solvent detergent treated IVIg, is manufactured by Octapharma Pharmazeutika (Vienna, Austria). First approved in 1995 in Germany as a 5 % liquid human immunoglobulin preparation and in subsequent years the Octagam^®^ 10 % formulation was available [27]. Octagam^®^ has been in clinical use for over 20 years in 80 countries. Approved indications differ between IVIg formulations and countries [13]. Globally, Octagam^®^ 5 % and 10 % are indicated for the treatment of primary and secondary immunodeficiencies, children with congenital AIDS and infection, Guillain–Barre Syndrome, Kawasaki Disease, allogenic bone marrow transplant, ITP and CIDP (5 % only) in the various global jurisdictions [13, 28]. Octagam^®^ has been shown to be a safe and efficacious therapy, it is well tolerated and not associated with significant treatment-related adverse reactions.

To date, one of the largest prospective studies on IVIg therapy evaluated the tolerability and safety of Octagam^®^ [16]. Commencing in 1995, data were collected from 310 study sites over a 10-year period. This study included 6357 subjects with various PIDs, SIDs, and autoimmune diseases, who received 92,958 infusions. Adverse drug reactions were reported in 4.2 % of patients, which corresponded to 0.35 % of all infusions [16]. Most of the adverse events recorded were mild (55.9 %) or moderate (34.3 %), and resolved quickly. The same study highlighted that there was no detectable relationship between the frequency of adverse drug reactions and elevated infusion rates or high dosages [16]. A recent prospective observational study in 117 PID patients compared the incidence of infusion-related adverse reactions of different IVIg products [29]. Of a total of 1765 infusions, the overall incidence of infusion-related adverse reactions was only 2.15, 92 % of which were classified as mild or moderate in severity. This study found that there were significantly fewer infusion-related adverse reactions for patients treated with Octagam^®^ (1.4 %) compared with another IVIg product (5.6 %) [29]. Of note, the incidence of adverse reactions reported in this study were surprisingly low, and this observation was thought to be attributed to infusion adjustments made for most patients prior to study start, an enrollment criteria that the patients be experienced (most patients had previous exposure to multiple brands that may have desensitized them), and inclusion of adverse reactions that occurred only during infusion. An additional limitation to the study was the manual regulation of infusion rates, due to the absence of infusion pumps [29].

The safety and quality of Octagam^®^ products is further ensured by the rigorous manufacturing process in which measures have been implemented to reduce the possible content of procoagulant factors. Testing of the final product also involves a thrombin generation assay that is designed to detect increased thromboembolic potential. Finally, to ensure viral safety is achieved—the manufacturing process involves two validated inactivation steps: a S/D method aimed at lipid enveloped virus inactivation, and a pH 4 treatment that reduces anti-complementary activation and aggregation of the IgG polymers. The S/D method is regarded by the World Hemophilia Federation as the “gold standard” in lipid enveloped virus inactivation [30]. This two-stage viral inactivation process has an excellent safety record [31], which is documented in the Octagam^®^ pharmacoviligence data (Octapharma, data on file).

### Canadian experience—Octagam^®^ 10 %

As of April 2013, Octagam^®^ 10 % became available to Canadian physicians and patients. Following approval in Germany in 1995, an open observations study was initiated to evaluate the tolerably and long-term safety profile of Octagam^®^ 5 % in routine clinical use in a wide variety of patients and diseases [16]. As the 10 % formulation is essentially similar to the less concentrated Octagam^®^ 5 %, it was expected to be equally efficacious, safe and well tolerated. We present here the results of the first Canadian retrospective file review that evaluated the routine clinical use of Octagam^®^ across three Canadian institutions: St. Paul’s Hospital in Vancouver British Columbia, St. Michael’s Hospital in Toronto Ontario, and St. Boniface Hospital in Winnipeg Manitoba.

## Methods

Data was collected over a 1-year period in an anonymous manner from IVIG request forms which were collected by the Blood Banks at St. Paul’s Hospital in Vancouver British Columbia, St. Michael’s Hospital in Toronto Ontario, and St. Boniface Hospital in Winnipeg Manitoba. The information included; the indications, the prescribing physicians’ specialty, and the number of patients treated for a given indication. A separate analysis was performed to determine the appropriateness of IVIg use based on various published Canadian Consensus Guidelines for IVIg utilization [7–10].

## Results

**Fig. 1:**
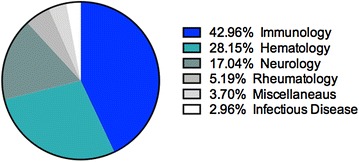
Percentage of patients treated with Octagam^®^ 10 % by indication category

Over a 1-year period, a total of 135 patients were treated with Octagam^®^ spanning 28 different indications (refer to Table 1). Similar to other IVIg products, Octagam^®^ was prescribed to patients with diseases spanning several indication groups: immunology, hematology, neurology, rheumatology, and infectious disease. The proportion of patients that received Octagam^®^ by indication is presented in Fig. 1. Octagam^®^ was most often prescribed to patients with diseases included in the immunology indication (PID, SID, acute antibody mediated rejection, desensitization for kidney transplantation, and secondary immune deficiency due to malignancy). Notably, 36/58 patients from this group were treated for PID. This was not surprising as two of the institutions involved have a dedicated immunology (immune deficiency) clinic. A large proportion of patients were also treated with Octagam^®^ for hematology indications, where 36/38 of these patients were diagnosed with ITP. A smaller proportion of patients treated were diagnosed with a neurological condition (23 patients, or 17 %)—however this included 11 different disease categories. A small proportion of patients were also treated for rheumatology and infection disease indications. A total of 5 patients (3.7 %) were not categorized by indication group, and this included patients treated for a pneumonia query tuberculosis, treatment post coronary artery bypass graft surgery, septic shock, and a dental/gum problem.

**Table 1:** Patients treated with Octagam^®^ 10 % across three Canadian institutions with corresponding indication group, and disease

Indication group	Disease	Number of patients	Total number of patients
Immunology	Primary immune deficiency	36	58
	Secondary immune deficiency	10	
	Acute antibody mediated rejection	6	
	Desensitization for kidney transplantation	4	
	Secondary immune deficiency due to malignancy	2	
Hematology	Idiopathic thrombocytopenic purpura	36	38
	Hemophagocytic lymphohistiocytosis	1	
	Pancytopenia/general weakness	1	
Neurology	Guillain-Barré syndrome	7	23
	Myasthenia Gravis	3	
	Chronic inflammatory demyelinating polyneuropathy	3	
	Stiff person syndrome	2	
	Acute disseminated encephalomyelitis	2	
	Multifocal motor neuropathy	1	
	Acute sensimotor polyneuropathy	1	
	Amyotrophic lateral sclerosis	1	
	Opsoclonus-myoclonus	1	
	Presumed neurosarcoidosis	1	
	Limbic encephalitis	1	
Rheumatology	Dermatomyositis	6	7
	Juvenile dermatomyositis	1	
Infectious disease	Group A streptococcus fasciitis	2	4
	Staphylococcal toxic shock	1	
	Cellulitis	1	
Miscellaneous	Pneumonia query tuberculosis, post coronary artery bypass graft surgery, septic shock, dental/gum problem	5	5

## Discussion

Globally, IVIg has been used in the treatment of approximately 100 different disease states and conditions. However, there is a paucity of compelling scientific evidence supporting many of these uses. Both the NAC and Ontario Regional Blood Coordinating Network (ORBCoN) monitor and evaluate IVIg utilization and related indications and these organizations provide useful guidance to practitioners regarding the appropriate use of IVIg [7, 8, 11, 12, 32].

ORBCoN recently published an audit of the routine clinical use of IVIg products across Ontario in 2012 [32]. The audit included 2246 patients over a 3-month period with diseases spanning 120 different indications. The patients were treated with one of the five IVIg products that were licensed in Canada at the time, and the labeled versus unlabeled utilization was examined. Labeled indications referred to those that were approved for use by Health Canada for at least one of the IVIg products. Using published clinical data, and Canadian IVIg guidelines [1, 7–10, 12, 18, 32] they summarized the off-label utilization (indications not approved by Health Canada) as either off-label and potentially indicated, or off-label and not indicated. Interestingly, their audit found that 55 % of utilization was for labeled indications. Approximately 33 % of IVIg utilization in Ontario is for unlabeled but potentially clinically appropriate conditions or diseases. This category includes disease states and conditions for which there are supporting data but that have not been evaluated by Health Canada. Examples of unlabeled but potentially appropriate uses for IVIg in Ontario include myasthenia gravis, juvenile dermatomyositis, pemphigus vulgaris and Guillain–Barre Syndrome. Lastly, 11 % of IVIg utilization was for off-label and not indicated. Examples of uses of IVIg in Ontario that are not considered fully supported by data but that are approved include kidney transplant, and hematopoietic stem cell transplant. For a comprehensive review of IVIg utilization and approved uses please consult ORBCoN 2012 Final Audit Report on IVIg [32].

**Fig. 2:**
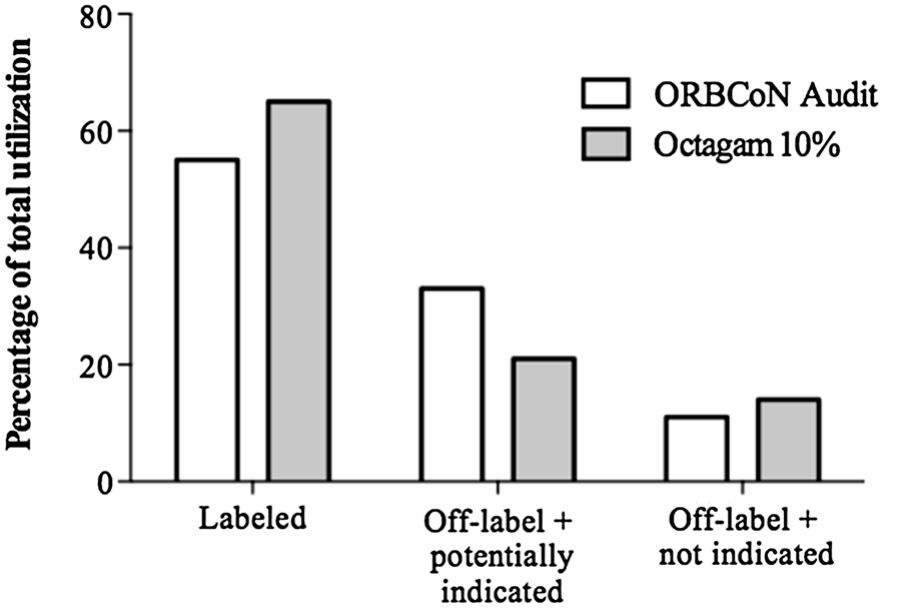
Comparison of the ORBCoN audit findings for IVIg utilization in Ontario to Octagam^®^ utilization across three Canadian Institutions highlighting labeled, off-label and potentially indicated, versus off-label and not indicated usage

In other words, 88 % of the utilization was considered to be appropriate. Applying the same criteria set forth in the ORBCoN audit and the Canadian IVIg guidelines, each of the indications identified in this study were similarly classified. Utilization of Octagam^®^ by three Canadian hospitals was found to be 65, 21, and 14 % for labeled (Health Canada has approved the indication for at least one IVIg product), off-label and potentially indicated, and off-label and not-indicated, respectively (Fig. 2). In other words, 86 % of the Octagam^®^ utilization was considered to be appropriate. These results suggest that Octagam^®^ is utilized in Canada like other IVIg products.

## Conclusion

According to the 2012 ORBCoN audit, the clinical specialties that most often prescribed IVIg were neurology, immunology and hematology, similar to the routine clinical use of Octagam^®^ [32]. There have been several initiatives in Canada that have been aimed at ensuring that IVIg utilization remains appropriate and in alignment with an evidenced-based approach to the practice of medicine. This retrospective review reported the routine clinical use and Canadian experience with Octagam^®^ in three hospitals over a 1-year period. Octagam^®^ was prescribed to patients across several disease indication groups including immunology, hematology, neurology, rheumatology and infection disease. When considering the Canadian IVIg guidelines, 116 of the 135 cases where Octagam^®^ was utilized were deemed to be appropriate [7–12, 32]. When compared to data from the 2012 ORBCoN Audit report of IVIg utilization across Ontario, this review demonstrated that Octagam^®^ utilization is similar to other IVIg products. Furthermore, these results suggest that Octagam^®^ has been integrated into the system and is prescribed in Canada like other IVIg products. This is not surprising since generally IVIg products have been utilized in a way that would suggest they have similar clinical efficacy and are interchangeable.

## Abbreviations

IVIg: intravenous immunoglobulin
PID: primary immune deficiency
SID: secondary immune deficiency
HIV: human immunodeficiency syndrome
ITP: idiopathic thrombocytopenic purpura
MMN: multifocal motor neuropathy
CLL: chronic B cell lymphocytic leukemia
CIDP: chronic inflammatory demyelinating polyneuropathy
NAC: National Advisory Committee on Blood and Blood Products
S/D: solvent/detergent
ORBCoN: Ontario Regional Blood Coordinating Network
